# More than one third of clinical practice guidelines on low back pain overlap in AGREE II appraisals. Research wasted?

**DOI:** 10.1186/s12874-022-01621-w

**Published:** 2022-07-05

**Authors:** Silvia Gianola, Silvia Bargeri, Michela Cinquini, Valerio Iannicelli, Roberto Meroni, Greta Castellini

**Affiliations:** 1 Unit of Clinical Epidemiology, IRCCS Istituto Ortopedico Galeazzi, Via R.Galeazzi 4, 20162 Milan, Italy; 2 Istituto di Ricerche Farmacologiche Mario Negri IRCCS, Milan, Italy; 3 IRCCS Istituto Ortopedico Galeazzi, Milan, Italy; 4 Department of Physiotherapy, LUNEX International University of Health, Differdange, Luxembourg; 5 Luxembourg Health & Sport Sciences Research Institute, Differdange, Luxembourg

**Keywords:** Practice guidelines, Methodological guideline appraisal, Methodological quality, AGREE instrument, Guidelines, Low back pain

## Abstract

**Background:**

Systematic reviews can apply the Appraisal of Guidelines for Research & Evaluation (AGREE) II tool to critically appraise clinical practice guidelines (CPGs) for treating low back pain (LBP); however, when appraisals differ in CPG quality rating, stakeholders, clinicians, and policy-makers will find it difficult to discern a unique judgement of CPG quality. We wanted to determine the proportion of overlapping CPGs for LBP in appraisals that applied AGREE II. We also compared inter-rater reliability and variability across appraisals.

**Methods:**

For this meta-epidemiological study we searched six databases for appraisals of CPGs for LBP. The general characteristics of the appraisals were collected; the unit of analysis was the CPG evaluated in each appraisal. The inter-rater reliability and the variability of AGREE II domain scores for overall assessment were measured using the intraclass correlation coefficient and descriptive statistics.

**Results:**

Overall, 43 CPGs out of 106 (40.6%) overlapped in seventeen appraisals. Half of the appraisals (53%) reported a protocol registration. Reporting of AGREE II assessment was heterogeneous and generally of poor quality: overall assessment 1 (overall CPG quality) was rated in 11 appraisals (64.7%) and overall assessment 2 (recommendation for use) in four (23.5%). Inter-rater reliability was substantial/perfect in 78.3% of overlapping CPGs. The domains with most variability were Domain 6 (mean interquartile range [IQR] 38.6), Domain 5 (mean IQR 28.9), and Domain 2 (mean IQR 27.7).

**Conclusions:**

More than one third of CPGs for LBP have been re-appraised in the last six years with CPGs quality confirmed in most assessments. Our findings suggest that before conducting a new appraisal, researchers should check systematic review registers for existing appraisals. Clinicians need to rely on updated CPGs of high quality and confirmed by perfect agreement in multiple appraisals.

**Trial Registration:**

Protocol Registration OSF: https://osf.io/rz7nh/

**Supplementary Information:**

The online version contains supplementary material available at 10.1186/s12874-022-01621-w.

## Introduction

Low back pain (LBP) is a major contributor to years lived with disability and a leading cause of limited activity and absence from work [1, 2]. In response to the global burden of LBP, major medical societies or specialized working groups have developed clinical practice guidelines (CPGs) for its diagnosis and management [3, 4]. The principles of CPGs design are well established but the growing multiplication of CPGs has cast doubt on their quality [5]. The current gold standard for the appraisal of CPG quality is the Appraisal of Guidelines for REsearch & Evaluation (AGREE) instrument developed by the AGREE Collaboration in 2003 [6–8]. The updated version, known as AGREE II, consists of 23 appraisal criteria (items) grouped into six independent quality domains. There are two overall assessment items: one to evaluate overall CPG quality (overall assessment 1) and one to judge whether a CPG should be recommended for use in practice (overall assessment 2) [9]. Substantial time and resources go into the development of CPGs ex novo, so it may be more efficient to adapt a high-quality CPG (or selected recommendations) for local use, when available [10–12]. Systematic reviews authors can apply AGREE II in their critical appraisal of CPGs for LBP [13–17], but stakeholders, clinicians, and policy makers may find it difficult to discern the highest quality CPG when appraisals give different quality ratings of overlapping CPGs. With this study we wanted to determine the proportion of CPGs evaluated in more than one appraisal (i.e., overlapping CPGs) and measure the inter-rater reliability (IRR) and variability of AGREE II scores for overlapping CPGs.

## Materials and methods

### Meta-epidemiological study

The study was conducted according to the guidelines for reporting meta-epidemiological methodology research [18] since the specific reporting checklist for methods research studies is currently under development (MethodologIcal STudy reporting Checklist [MISTIC]) [19]. The protocol is available on the public Open Science Framework (OSF) repository at https://osf.io/rz7nh/

### Search strategy and study selection

We summarized the findings of systematic reviews that applied the AGREE II tool to appraise the quality of CPGs for LBP. We defined these systematic reviews as “appraisals”. For details about the AGREE II instrument, see https://www.agreetrust.org/resource-centre/agree-ii/.

We systematically searched six databases (PubMed, EMBASE, CINAHL, Web of Science, Psychinfo, PEDRO) from January 1, 2010 through March 3, 2021. AGREE II was published in 2010 [6]. The full search strategy is presented in Additional file 1.

### Eligibility criteria

Two independent reviewers screened titles and abstracts against eligibility criteria: 1) systematic reviews (i.e., CPGs appraisals) that used the AGREE II tool to evaluate CPGs quality; 2) CPGs for LBP prevention, diagnosis, management, and treatment irrespective of cause (e.g., non-specific LBP, spondylolisthesis, lumbar stenosis, radiculopathy); 3) AGREE II ratings were reported. Included in the present study were appraisals on mixed populations (e.g., neck and back pain) when the data on back pain were reported separately. A third reviewer was consulted to resolve reviewer disagreement. Rayyan software [20] was used to manage screening and selection.

### Data extraction

Data were entered on a pre-defined data extraction form (Excel spreadsheet). Two authors extracted the data for: study author, year of publication, protocol registration, number of raters, training in use of the AGREE II tool, population, intervention, exclusion criteria for each appraisal, references of CPGs, AGREE II items/domain scores and two overall assessments: overall assessment 1 (overall CPG quality [measured on a 1-7 scale]) and overall assessment 2 (recommendation for use [yes, yes with modifications, no]). When reported by the appraisers, quality ratings (high, moderate, low) were also extracted.

The reporting of overall assessment varied across appraisals. For overall assessment 2, we collected information about the number of raters who selected the categories “yes”, “yes with modification” or “no” (e.g., 75% raters judged “yes”; 25% “yes with modifications” and 0% “no”) labeling this “raw recommendation for use”. In appraisals that reported only a single recommendation (such as yes) without the percentage for all three categories, we assigned this category by default, labeling this “final recommendation for use” [21].

The corresponding authors were contacted when AGREE II domain scores and overall assessments were not reported. When no response was received, we calculated the domain scores based on AGREE II item scores according to the AGREE II formulas [9].

### Data synthesis and analysis

The characteristics of the appraisals eligible for inclusion were summarized using descriptive statistics. Overlapping was defined as how many times a CPG was re-assessed for quality in different appraisals using the AGREE II tool. We measured IRR and variability of the AGREE II domain scores for CPGs that were assessed by at least three appraisals. We used the average intraclass correlation coefficient (ICC) with 95% confidence interval (CI) of the six domain scores to formulate agreement between overlapping CPGs [22]. The degree of agreement was graded according to Landis and Koch [23]: slight (0.01-0.2); fair (0.21-0.4); moderate (0.41-0.6); substantial (0.61-0.8); and almost perfect (0.81-1). For quantitative variables (AGREE II domain scores and overall assessment 1), we measured variability by calculating the interquartile range (IQR) as the difference between the first and the third quartile (Q3-Q1). We measured variability in qualitative variables (overall assessment 2 and quality ratings) as agreement/disagreement of judgments. We defined “perfect agreement” when all appraisals gave the same judgment for the same category (e.g., all judged “high quality” for the same CPGs, IRR=1). Variability of each of the six domain scores for the overlapping CPGs (assessed by at least three appraisals) is reported as mean IQR. Statistical significance was set at *P* < 0.05. All tests were two-sided. Data analysis was performed using STATA [24].

## Results

### Search results

**Fig. 1:**
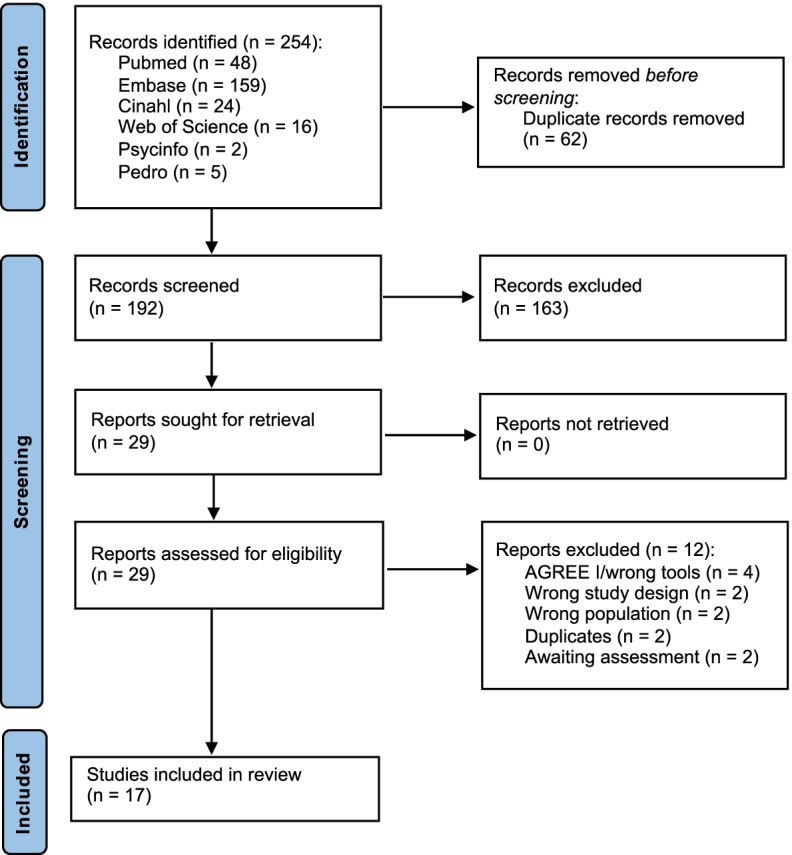
Study flow chart selection

The systematic search retrieved 254 records. After duplicates were removed, 192 records were obtained, 163 of which were discarded. The full text of the remaining 29 was examined; 12 did not meet the inclusion criteria (Fig. 1). Finally, 17 appraisals that applied the AGREE II tool were included in the analysis [17, 25–40].

### Characteristics of CPG appraisals

Table 1 presents the general characteristics of the 17 appraisals. The median year of publication was 2020 (range, 2015-2021). Eleven appraisals assessed CPGs for LBP and six assessed CPGs not restricted to LBP alone (e.g., chronic musculoskeletal pain). Seven appraisals (41.2%) reported a protocol registration in PROSPERO and two (11.8%) a protocol registration in other online registries or repositories. Three appraisals (17.6%) involved four AGREE II raters and the remaining involved two or three. Six appraisals (35.3%) stated that the raters had received training for using the tool. The rating of all six domains was reported in 14 appraisals (82.4%) [17, 25–35, 37, 40] and the rating of 23 item scores in two [36, 39]. Overall assessment 1 (overall CPG quality) was reported in 11 appraisals (64.7%) [25–30, 32–34, 37, 40] and overall assessment 2 (recommendation for use) in four (23.5%) [25, 27, 28, 32]. A quality rating (not part of the AGREE II tool) was given in nine appraisals (53%) [17, 26, 28, 29, 33, 34, 36, 37, 39]. One appraisal reported AGREE II ratings in supplementary materials that were unavailable [38]. Four authors [29, 35–37] supplied missing data as requested.

**Table 1:** General characteristics of appraisals

**APPRAISAL**	**PROTOCOL PLANNED**	**SEARCH DATE**	**NO. OF RATERS**	**NO. OF CPG**	**POPULATION**	**INTERVENTION**
**ACEVEDO 2016 **[25]	No	2000 - 2014	2	5	Chronic LBP	Interventional management (surgical and non-surgical)
**ANDERSON 2021 **[26]	PROSPERO	Inception - January 2020	2	10	Lumbar spinal stenosis	Diagnosis, treatment, management
**CASTELLINI 2020 **[27]	PROSPERO	January 2016 - January 2020	4	21	LBP	Rehabilitation, pharmacological or surgical therapy
**CORP 2021 **[28]	Online repository	January 2013 - May 2020	1-2	12	Adults with neck pain and LBP including whiplash-related disorders or symptoms of radiculopathy (e.g., radicular pain)	Treatment deliverable via primary care or referral pathways to secondary care
**DONISELLI 2018 **[29]	No	2009 - March 2017	4	8	LBP	Assessment and management
**ERNSTZEN 2017 **[30]	PROSPERO	2000 - May 2015	3	12	Adults with chronic MSK pain including LBP	Evaluation, diagnosis, and management of chronic MSK pain
**FRANZ 2015 **[31]	No	2000 - June 2014	NR	2	LBP	Diagnosis and treatment
**HOYDONCKX 2020 **[32]	No	January 2008 - December 2018	3	2	Adults with chronic pain including LBP	Diagnosis and treatment
**KRENN 2020 **[33]	No	2013 - September 2020	2	10	People, any age and sex, with specific-LBP	Diagnosis and/or treatment
**LIN 2020 **[34]	PROSPERO	2011 - 2017	3	15	Adults with spinal pain (lumbar, thoracic, cervical spine), hip/knee pain including hip/knee OA and shoulder pain	Assessment and treatment
**MERONI 2019 **[17]	No	January 2011 - December 2019	3	10	Non-specific chronic LBP	Clinical management of nonspecific chronic LBP in primary care
**NG 2021 **[35]	PROSPERO^b^	2008 – October 2018	2-3	22	Adults with any type of LBP	Treatment and/or management of LBP
**NORDIN 2018 **[36]	PROSPERO	2010 - October 2017	2	15	Adults and children with neck pain and associated disorders, mechanical thoracic spinal pain, musculoskeletal chest pain, non-specific LBP with or without radiculopathy, infection associated with the spine (i.e., bacteria, fungi), spinal deformity (kyphosis, lordosis, scoliosis), myelopathy, and inflammatory arthritis	Assessment and intervention in differential diagnosis
**RATHBONE 2020 **[37]	Online repository	1946 - March 2020	2	36	Adult population with primary LBP	Within the scope of physiotherapy
**STANDER 2020**^a^ [38]	No	Inception - January 2019	NR	3	Adults with acute or subacute LBP	Physiotherapy assessment and management
**WONG 2017 **[39]	PROSPERO	January 2005 - April 2014	2	13	Adults and/or children with LBP with or without radiculopathy;	Therapeutic noninvasive management
**YAMAN 2015 **[40]	No	Search done in July 2014	4	3	NASS evidence-based clinical practice CPGs	-

*LBP* Low back pain, *CPG* Clinical practice guideline, *MSK* Musculoskeletal, *NASS* North American Spine Society, *NR* Not reported

^a^qualitative synthesis due to missing data

^b^information found in the previous publication [41]

### Overlapping CPGs

A total of 43/106 CPGs (40.6%) were overlapping in 17 appraisals (i.e., assessed by at least two appraisals) and 23 CPGs [42–65] had been assessed by at least three appraisals. The six CPGs that most often overlapped were issued by: the National Institute for Health and Care Excellence (NICE) 2016 [63] (9 appraisals), the American College of Physicians (ACP) 2017 [47] (8 appraisals), the American Physical Therapy Association (APTA) 2012 [56] (8 appraisals), the Belgian Health Care Knowledge Centre (KCE) 2017 [43] (6 appraisals), the American Pain Society (APS) 2009 [65] (5 appraisals), and the Council on Chiropractic Guidelines and Practice Parameters (CCGPP) 2016 [55] (5 appraisals). Table 2 presents the overlapping CPGs.12874_1621

**Table 2:** Overlapping CPGs for LBP

	**No. of CPGs (out of 106)**	**%**	**ORGANISATION ACRONYM**	**YEAR**	**COUNTRY**	**AUTHOR**
**Overlapping 9 times**	1	0.9	NICE	2016	UK	Arvin and De campos [63]
**Overlapping 8 times**	2	1.9	ACP	2017	USA	Qaseem[47]
			APTA	2012	USA	Delitto [56]
**Overlapping 7 times**	0	0.0	-	-	-	-
**Overlapping 6 times**	1	0.9	KCE	2017	Belgium	van Wambeke [43]
**Overlapping 5 times**	2	1.9	APS	2009	USA	Chou [65]
			CCGPP	2016	USA	Globe [55]
**Overlapping 4 times**	4	3.8	ASIPP	2013	USA	Manchikanti [49]
			KNGF	2013	Netherlands	Staal [46]
			TOP	2017	Canada	Low Back Pain Working Group [53]
			VA/DoD	2017	USA	Pangarkar [48]
**Overlapping 3 times**	13	12.3	CAAM	2016	China	Zhao [42]
			CCGI	2018	Canada	Bussières [60]
			Cheng	2012	JAPAN	Cheng [59]
			DAI	2017	Germany	Chenot [58]
			DHA	2017	Denmark	Stochkendahl [45]
			ICSI	2012	USA	Goertz [54]
			ICSI	2018	USA	Thorson [44]
			NASS	2013	USA	Kreiner [50]
			NASS	2014	USA	Kreiner [51]
			NICE	2009	UK	Savigny [64]
			OMG	2012	Canada	Brosseau L[61]
			PSP	2017	Poland	Kassolik [52]
			SIGN	2013	UK	NR [62]
**Overlapping 2 times**	20	18.9	ACOEM	2019	USA	Hegmann [66]
			ACOEM	2016	USA	Hegmann[67]
			ACR	2016	USA	Patel [68]
			AOA	2016	USA	Task Force on the Low Back Pain Clinical Practice Guidelines [69]
			APS	2007	USA	Chou [70]
			BPS	2013	UK	Lee [71]
			DSA	2016	Netherlands	Itz [72]
			-	2006	Europe	Airaksinen [73]
			Institute of Medicine	2019	USA	Deer [74]
			KIOM	2017	Korea	Jun [75]
			NASS	2012	USA	Kreiner [76]
			NASS	2014	USA	Kreiner [77]
			NASS	2016	USA	Matz [78]
			NVL	2017	Germany	Bundesärztekammer [79]
			PARM	2012	Philippine	NR [80]
			-	2016	Italy	Picelli [81]
			SOECGP	2011	USA	Livingstone [82]
			TOP	2015	Canada	TOP [83]
			TOP	2009	Canada	TOP [84]
			University of Michigan	2011	USA	Chiodo [85]
**Overall (>=2)**	**43**	**40.6**				

*ACOEM* American College of Occupational and Environmental Medicine, *ACP* American College of Physicians, *ACR* American College of Radiology, *AOA* American Osteopathic Association, *APS* American Pain Society, *APTA* American Physical Therapy Association, *ASIPP* American Society of Interventional Pain Physicians, *BPS* British Pain Society, *CAAM* China Association of Acupuncture-Moxibustion, *CCGI* Canadian Chiropractic Guideline Initiative, *CCGPP* Council on Chiropractic Guidelines and Practice Parameters, *DAI* Deutsches Ärzteblatt International, *DHA* Danish Health Authority, *DSA* Dutch Society of Anesthesiologists, *ICSI* Institute for Clinical Systems Improvement, *KCE* Belgian Health Care Knowledge Centre, *KIOM* Korea Institute of Oriental Medicine, *KNGF* Koninklijk Nederlands Genootschap voor Fysiotherapie, *NASS* North American Spine Society, *NICE* National Institute for Health and Care Excellence, *NVL* Nationale Versorgungs Leitlinie, *OMG* Ottawa Methods Group, *PARM* Philippine Academy of Rehabilitation Medicine, *PSP* Polish Society of Physiotherapy, *SIGN* Scottish Intercollegiate Guidelines Network, *SOEGCP* State of Oregon Evidence-based Clinical Guidelines Project, *TOP* Toward Optimized Practice Low Back Pain Working Group, *VADoD* Veterans Affairs/Department of Defense Collaboration Office

### Inter-rater reliability

Table 3 presents the ICC averages of the overlapping CPGs assessed by at least three appraisals**.** IRR was perfect in 13 CPG ratings (56.6%), substantial in five (21.7%), moderate in two (8.7%), fair in one (4.3%), and slight in two (8.7%). The highest agreement was reached in the ACP 2017 [47], the APTA 2012 [56], and the APS 2009 [65] and the lowest in the NICE 2009 [64], the Toward Optimized Practice Low Back Pain Working Group (TOP) 2017 [53], and the American Society of Interventional Pain Physicians (ASIPP) 2013 [49]. In the most often overlapping CPGs (at least five appraisals), the IRR was perfect in all, except the KCE 2017 [43] (substantial).

**Table 3:** ICC of overlapping CPGs assessed by at least three appraisals

**CPG**	**ICC AVERAGE**	**CI LOWER**	**CI UPPER**	**ICC INDIVIDUAL**	**CI LOWER**	**CI UPPER**	**AGREEMENT**
**ACP 2017 **[47]	0.98	0.95	1.00	0.89	0.72	0.98	Perfect
**APTA 2012 **[56]	0.96	0.90	0.99	0.77	0.52	0.96	Perfect
**APS 2009 **[65]	0.96	0.86	0.99	0.82	0.56	0.97	Perfect
**ICSI 2018 **[44]	0.95	0.77	0.99	0.86	0.53	0.98	Perfect
**CAAM 2016 **[42]	0.92	0.70	0.99	0.8	0.44	0.97	Perfect
**VADOD 2017 **[48]	0.92	0.72	0.99	0.74	0.4	0.95	Perfect
**DHA 2017 **[45]	0.9	0.61	0.98	0.75	0.34	0.95	Perfect
**KNGF 2013 **[46]	0.89	0.6	0.98	0.66	0.27	0.93	Perfect
**OMG 2012 **[61]	0.89	0.56	0.98	0.72	0.3	0.95	Perfect
**CCGPP 2016 **[55]	0.85	0.55	0.98	0.54	0.2	0.89	Perfect
**NICE 2016 **[63]	0.84	0.54	0.97	0.39	0.13	0.82	Perfect
**CHENG 2012 **[59]	0.83	0.31	0.97	0.61	0.13	0.92	Perfect
**NASS 2014 **[51]	0.82	0.33	0.97	0.6	0.14	0.92	Perfect
**NASS 2013 **[50]	0.70	0.03	0.95	0.43	0.01	0.86	Substantial
**ICSI 2012 **[54]	0.68	0.01	0.95	0.42	0,00	0.86	Substantial
**KCE 2017 **[43]	0.66	0.19	0.94	0.25	0.04	0.72	Substantial
**SIGN 2013 **[62]	0.62	0.00	0.94	0.36	-0.13	0.85	Substantial
**PSP 2017 **[52]	0.62	0.00	0.93	0.35	-0.01	0.82	Substantial
**CCGI 2018 **[60]	0.54	0.00	0.92	0.28	-0.06	0.79	Moderate
**DAI 2017 **[58]	0.46	0.00	0.89	0.22	-0.03	0.72	Moderate
**NICE 2009 **[64]	0.3	0.00	0.85	0.13	-0.09	0.65	Fair
**TOP 2017 **[53]	0.16	0.00	0.81	0.06	-0.14	0.59	Slight
**ASIPP 2013 **[49]	0.14	0.00	0.88	0.04	-0.25	0.65	Slight

*ACP* American College of Physicians, *APS* American Pain Society, *APTA* American Physical Therapy Association, *ASIPP* American Society of Interventional Pain Physicians, *CAAM* China Association of Acupuncture-Moxibustion, *CCGI* Canadian Chiropractic Guideline Initiative, *CCGPP* Council on Chiropractic Guidelines and Practice Parameters, *CPG* Clinical Practice Guideline, *DAI* Deutsches Ärzteblatt International, *DHA* Danish Health Authority, *ICC* Intraclass Correlation Coefficient, *ICSI* Institute for Clinical Systems Improvement, *KCE* Belgian Health Care Knowledge Centre, *KNGF* Koninklijk Nederlands Genootschap voor Fysiotherapie, *NASS* North American Spine Society, *NICE* National Institute for Health and Care Excellence, *OMG* Ottawa Methods Group, *PSP* Polish Society of Physiotherapy, *SIGN* Scottish Intercollegiate Guidelines Network, *TOP* Toward Optimized Practice Low Back Pain Working Group, *VADoD* Veterans Affairs/Department of Defense Collaboration Office

### Variability in domain scores

**Fig. 2:**
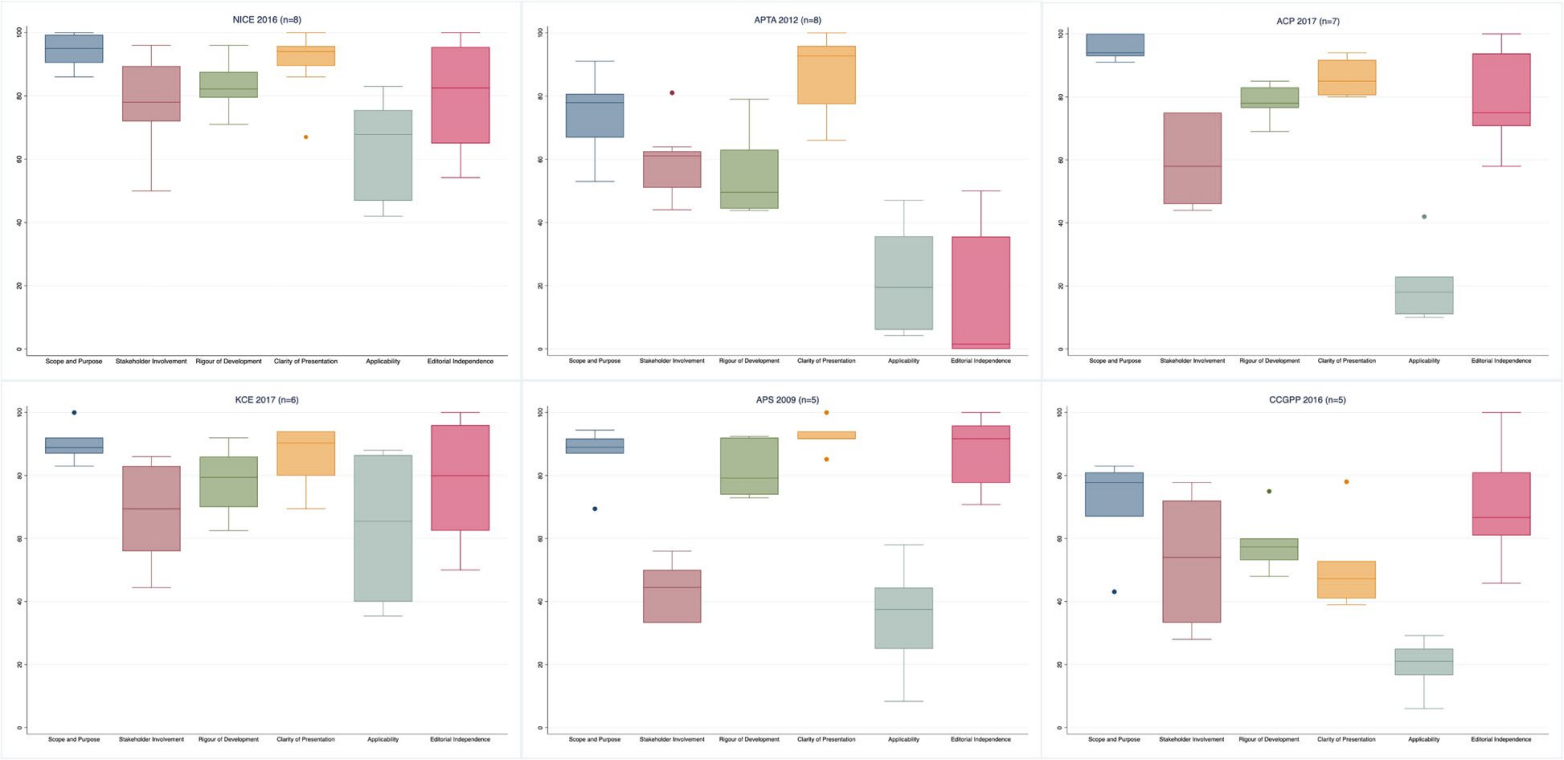
Variability of six domains of AGREE II applied to the most often overlapping CPGs (assessed by at least five appraisals). The vertical axis represents AGREE II domain scores (0-100), the horizontal axis represents six AGREE II Domains. Legend. Domain 1: Scope and Purpose, Domain 2: Stakeholder involvement, Domain 3: Rigour of Development, Domain 4: Clarity of presentation, Domain 5: Applicability, Domain 6: Editorial Independence. ACP: American College of Physicians; APS: American Pain Society; APTA: American Physical Therapy Association; CCGPP: Council on Chiropractic Guidelines and Practice Parameters; KCE: Belgian Health Care Knowledge Centre; NICE: National Institute for Health and Care Excellence. * NICE 2016 was assessed by nine appraisals but the domain scores were available for eight; ACP 2017 was assessed by eight appraisals but available for seven

The most variable domains of overlapping CPGs (assessed by at least three appraisals) were Domain 6 - Editorial Independence (mean IQR 38.6), Domain 5 - Applicability (mean IQR 28.9), and Domain 2 - Stakeholder Involvement (mean IQR 27.7). Among all domains, the most variable CPG was issued by TOP 2017 [53] (mean IQR 51.4) and the least was issued by the Institute for Clinical Systems Improvement (ICSI) 2018 [44] (mean IQR 11) (Table 4). Domain 6 – Editorial Independence was the most variable domain of the CPGs that most often overlapped (assessed by at least five appraisals) (Fig. 2).

**Table 4:** Domain score variability of overlapping CPGs assessed by at least three appraisals

**CPG**	**D1 (IQR)**	**D2 (IQR)**	**D3 (IQR)**	**D4 (IQR)**	**D5 (IQR)**	**D6 (IQR)**	**Mean**
**ICSI 2018 **[44]	8	5.8	12.2	5.1	18	16.8	11
**ACP 2017 **[47]	7	29	6.4	11.1	11.9	23	14.7
**CCGI 2018 **[60]	3.7	26.7	1	21.3	30	17	16.6
**CAAM 2016 **[42]	5.5	8.8	14.9	50	6.3	20.8	17.7
**SIGN 2013 **[62]	15	11.1	19	20	18	27	18.4
**DHA 2017 **[45]	8	32	13	8	29	24	19
**NICE 2016 **[63]	9.9	22.1	9.6	8.6	30.9	34.7	19.3
**APS 2009 **[65]	14.9	19.7	18.8	8.6	34.5	23.7	20
**OMG 2012 **[61]	5.7	17.1	20.3	66.9	21	12.3	23.9
**APTA 2012 **[56]	20.5	12.7	23.9	24.3	33.7	36.8	25.3
**VaDod 2017 **[48]	12.7	19.7	25.8	12.1	29.5	52.1	25.3
**ICSI 2012 **[54]	22	37.3	33	16.9	14	33	26
**KCE 2017 **[43]	8	30.7	19.4	16.6	48	37.6	26.7
**Cheng 2012 **[59]	53.4	35	17.8	44.3	12.2	0	27.1
**CCGPP 2016 **[55]	27	44.2	16.9	25.4	15.8	37.1	27.7
**NASS 2014 **[51]	14	25	21.8	49	6.7	70.3	31.1
**KNGF 2013 **[46]	28.5	7.2	56.1	27	37.9	37.5	32.4
**NICE 2009 **[64]	8.3	22.2	37.5	11.1	47.9	70.8	33
**ASIPP 2013 **[49]	18.1	21	21.3	32.9	33.9	78.1	34.2
**NASS 2013 **[50]	13	45	35	50	56	54	42.2
**PSP 2017 **[52]	52.8	53	12.4	63.6	42	41.7	44.2
**DAI 2017 **[58]	71	56	43	33	36	41.7	46.8
**TOP 2017 **[53]	24.6	56	66	14.2	50.9	97	51.4
**Mean**	19.6	27.7	23.7	27	28.9	38.6	-

Variability is expressed as the IQR (quartile 3-quartile 1) of domain scores for overlapping CPGs. D, domain. Domain 1: Scope and Purpose, Domain 2: Stakeholder involvement, Domain 3: Rigour of Development, Domain 4: Clarity of presentation, Domain 5: Applicability, Domain 6: Editorial Independence

*ACP* American College of Physicians, *APS* American Pain Society, *APTA* American Physical Therapy Association, *ASIPP* American Society of Interventional Pain Physicians, *CAAM* China Association of Acupuncture-Moxibustion, *CCGI* Canadian Chiropractic Guideline Initiative, *CCGPP* Council on Chiropractic Guidelines and Practice Parameters, *DAI* Deutsches Ärzteblatt International, *DHA* Danish Health Authority, *ICSI* Institute for Clinical Systems Improvement, *KCE* Belgian Health Care Knowledge Centre, *KNGF* Koninklijk Nederlands Genootschap voor Fysiotherapie, *NASS* North American Spine Society, *NICE* National Institute for Health and Care Excellence, *OMG* Ottawa Methods Group, *PSP* Polish Society of Physiotherapy, *SIGN* Scottish Intercollegiate Guidelines Network, *TOP* Toward Optimized Practice Low Back Pain Working Group, *VADoD* Veterans Affairs/Department of Defense Collaboration Office

### Variability of overall assessments 1 and 2

Because of missing data and heterogeneity of reporting (e.g., 0-100 scale or 1-7 scale for overall assessment 1; raw recommendation for use or final recommendation for overall assessment 2), we trasparently reported the judgments of the two overall assessments of overlapping CPGs assessed by at least three appraisals in Table 5. For overall assessment 2, a perfect agreement was achieved  in 5/20 CPG assessments (25%), heterogeneity of reporting in 8/20 (40%), and no complete agreement in 7/20 (35%). For quality ratings (high, moderate, low), a perfect agreement was achieved in 10/19 (53%) while the remaining 9/10 (47%) did not completely agree.

**Table 5:** AGREE II overall assessment of CPGs assessed by at least three appraisals

APPRAISAL AUTHOR	OA1	VARIABILITY OA1	VARIABILITY OA1	OA2	AGREEMENT OA2	QUALITY RATING	AGREEMENT QUALITY RATING
		(0-100 scale)	(1-7 scale)				
		Median (Q1-Q3)	Median (Q1-Q3)				
**NICE 2016** [63]							
**Castellini 2020 **[27]	95,83	88 (83-92.4)	6 (5.5-7)	Yes	Heterogeneity of reporting		Perfect agreement
**Corp 2021** [28]	83			Yes			
**Doniselli 2018** [29]	83			Yes (3 raters); Maybe (1 rater)^a^			
**Krenn 2020** [33]	7					High	
**Lin 2020** [34]	89					High	
**Meroni 2019** [17]	88						
**Ng 2021** [35]	6^ab^			Yes (1 rater); Yes with modifications (1 rater)^a^			
**Rathbone 2020** [37]	5,5			Yes^a^		High	
**Stander 2020** [38]							
**ACP 2017** [47]							
**Castellini 2020** [27]	75	77 (68.3-82)	5 (5-5.5)	Yes	Heterogeneity of reporting		No agreement
**Doniselli 2018** [29]	79			Yes (2 raters); Maybe (1 rater)^a^			
**Krenn 2020** [33]	5					Moderate	
**Lin 2020** [34]	83					Low	
**Meroni 2019** [17]	66						
**Ng 2021** [35]	5,5^ab^			Yes with modifications^a^			
**Rathbone 2020** [37]	5			Yes with modifications^a^		Average	
**Stander 2020** [38]							
**APS 2009** [65]							
**Acevedo 2016** [25]	6	na	5.6 (5.1-6.0)	Yes	No agreement		Perfect agreement
**Anderson 2021** [26]	5,9					Satisfactory	
**Ng 2021** [35]	5^ab^			Yes with modifications^a^			
**Wong 2016** [39]						High	
**Hoydonckx 2020** [32]	5,33						
**APTA 2012** [56]							
**Doniselli 2018** [29]	67	55 (44-67)	4.8	Yes (2 raters); No (2 raters)^a^	Heterogeneity of reporting		Perfect agreement
**Franz 2015** [31]							
**Lin 2020** [34]	44					Low	
**Meroni 2019** [17]	55						
**Ng 2021** [35]	5^ab^			Yes with modifications^a^			
**Rathbone 2020** [37]	4,5			Yes with modifications^a^		Low	
**Wong 2016** [39]						Low	
**Nordin 2018** [36]							
**KCE 2017** [43]							
**Castellini 2020** [27]	83,33	83.3 (61-100)	5 (4.5-6)	Yes	No agreement		No agreement
**Corp 2021** [28]	100			Yes		High	
**Krenn 2020** [33]	4,5					Moderate	
**Lin 2020** [34]	61					High	
**Ng 2021** [35]	5^ab^			Yes with modifications^a^			
**Rathbone 2020** [37]	6			Yes^a^		High	
**CCGPP 2016** [55]							
**Castellini 2020** [27]	29,17	44 (29.2-47)	na	No	Heterogeneity of reporting		Perfect agreement
**Lin 2020** [34]	44					High	
**Meroni 2019** [17]	47						
**Ng 2021** [35]	4,5^ab^			Yes with modifications^a^			
**Rathbone 2020** [37]	5,5			Yes (1 rater); Yes with modifications (1 rater)^a^		High	
**ASIPP 2013** [49]							
**Acevedo 2016** [25]	5		5.6 (5-5.6)	No	Heterogeneity of reporting		No agreement
**Anderson 2021** [27]	5,6					Not satisfactory	
**Hoydinckx 2020** [33]	5,66			Yes (1 rater); Yes with modifications (1 rater); No (1 rater)			
**Nordin 2018** [36]	75^a^			Yes^a^		High	
**CAAM 2016** [42]							
**Castellini 2020** [27]	45,8	na	na	No	No agreement		na
**Ng 2021** [35]	4^ab^			Yes with modifications^a^			
**Rathbone 2020** [37]	2,5			Yes with modifications^a^		Low	
**CCGI 2018** [60]							
**Castellini 2020** [27]	87,5	na	na	Yes	Heterogeneity of reporting		Perfect agreement
**Krenn 2020** [33]	6					High	
**Rathbone 2020** [37]	6			Yes (1 rater); Yes with modifications (1 rater)^a^		High	
**Cheng 2012** [59]							
**Lin 2020** [34]	17	na	na		Perfect agreement	Low	Perfect agreement
**Ng 2021** [35]	4^ab^			Yes with modifications^a^			
**Rathbone 2020** [37]	4			Yes with modifications^a^		Low	
**DAI 2017** [58]							
**Meroni 2019** [17]	80	na	na		Perfect agreement	Excellent	No agreement
**Ng 2021** [35]	4^ab^			Yes with modifications^a^			
**Rathbone 2020** [37]	3			Yes with modifications^a^		Low	
**DHA 2017** [45]							
**Doniselli 2018** [30]	92	na	na	Yes (3 raters); Maybe (1 rater)^a^	Heterogeneity of reporting		No agreement
**Lin 2020** [34]	67					High	
**Rathbone 2020** [37]	4,5			Yes with modifications^a^		Average	
**ICSI 2012** [54]							
**Doniselli 2018** [30]	79	na	na	Yes (2 raters); Maybe (2 raters)^a^	Heterogeneity of reporting		na
**Lin 2020** [34]	56					Low	
**Ng 2021** [35]	4,5^ab^			Yes with modifications^a^			
**ICSI 2018** [44]							
**Castellini 2020** [27]	62,5			Yes, with modifications	No agreement		Perfect agreement
**Krenn 2020** [33]	5,5	na	na			Moderate	
**Rathbone 2020** [37]	5,5			Yes^a^		Average	
**KNGF 2013** [46]							
**Franz 2015** [31]		na	na		Perfect agreement		na
**Meroni 2019** [17]	43						
**Ng 2021** [35]	3^ab^			No^a^			
**Rathbone 2020** [37]	3			No^a^		Average	
**NASS 2013** [50]							
**Anderson 2021** [27]	5,5	na	na		na	Satisfactory quality	No agreement
**Lin 2020** [34]	39					Low	
**Rathbone 2020** [37]	4			Yes with modifications^a^		Low	
**NASS 2014** [51]							
**Lin 2020** [34]	39	na	na		na	Low	No agreement
**Rathbone 2020** [37]	4			Yes with modifications^a^		Low	
**Wong 2016** [39]						High	
**NICE 2009** [64]							
**Acevedo 2016** [25]	6,5	na	na	Yes	No agreement		na
**Ng 2021 2021** [35]	4,5^ab^			Yes with modifications^a^			
**Wong 2016** [39]						High	
**OMG 2012** [61]							
**Ng 2021 2021** [35]	4,5^ab^	na	na	Yes with modifications^a^	Perfect agreement		Perfect agreement
**Rathbone 2020** [37]	4			Yes with modifications^a^		Low	
**Wong 2016** [39]						Low	
**PSP 2017** [52]							
**Castellini 2020** [27]	4,17	na	na	No	No agreement		No agreement
**Corp 2021** [28]	33			No		Low	
**Rathbone 2020** [37]	4			Yes with modifications^a^		Average	
**SIGN 2013** [62]							
**Ernstzen 2017** [30]	6,5	na	na		na		Perfect agreement
**Meroni 2019** [17]	81					Excellent	
**Wong 2016** [39]						High	
**TOP 2017** [53]							
**Castellini 2020** [27]	58,33	na	na	No	No agreement		No agreement
**Meroni 2019** [17]	89					Excellent	
**Rathbone 2020** [37]	3,5			Yes with modifications^a^		Low	
**VA/DoD 2017** [48]							
**Castellini 2020** [27]	70,83	na	na	Yes, with modifications	Perfect agreement		Perfect agreement
**Kreen 2020** [33]	5,5					Moderate	
**Meroni 2019** [17]	67						
**Rathbone 2020** [37]	4			Yes with modifications^a^		Average	

OA1, overall assessment 1; OA2, overall assessment 2. Empty cells indicate judgements not reported

^a^data sent

^b^mean judgement between raters. Na, not assessed due to missing data

*ACP* American College of Physicians, *APS* American Pain Society, *APTA* American Physical Therapy Association, *ASIPP* American Society of Interventional Pain Physicians, *CAAM* China Association of Acupuncture-Moxibustion, *CCGI* Canadian Chiropractic Guideline Initiative, *CCGPP* Council on Chiropractic Guidelines and Practice Parameters, *DAI* Deutsches Ärzteblatt International, *DHA* Danish Health Authority, *ICSI* Institute for Clinical Systems Improvement, *KCE* Belgian Health Care Knowledge Centre, *KNGF* Koninklijk Nederlands Genootschap voor Fysiotherapie, *na* not assessed, *NASS* North American Spine Society, *NICE* National Institute for Health and Care Excellence, *OMG* Ottawa Methods Group, *PSP* Polish Society of Physiotherapy, *SIGN* Scottish Intercollegiate Guidelines Network, *TOP* Toward Optimized Practice Low Back Pain Working Group, *VADoD* Veterans Affairs/Department of Defense Collaboration Office

Table 6 presents the variability in the most often overlapping CPGs (assessed by at least 5 appraisals) Overall assessment 1 varied the most in the KCE 2017 [43] (IQR 23 on a 0-100 scale) and the least in the NICE 2016 [63] (IQR 9.4 on a 0-100 scale). Agreement in quality ratings was perfect in the NICE 2016 [63] (3/3 high quality), the APTA 2012 [56] (3/3 low quality), and the CCGPP 2016 [55] (2/2 high quality).

**Table 6:** AGREE II overall assessment of the most often overlapping CPGs (assessed by at least five appraisals)

**CPG**	**OA 1 (0-100 scale) Median (Q1-Q3) (No. of available assessments)**	**OA 1 (1-7 scale) Median (Q1-Q3) (No. of available assessments)**	**OA 2 No. of overall ratings**^a^	**Quality rating No. of overall ratings**
**NICE 2016 **[63]	88 (83-92.4) (*n*=5)	6 (5.5-7) (*n*=3)	3/5 Yes 2/5 Raw	3/3 High
**ACP 2017 **[47]	77 (68.3-82) (*n*=4)	5 (5-5.5) (*n*=3)	1/4 Yes 2/4 Yes with modifications 1/4 Raw	2/3 Moderate 1/3 Low
**APS 2009 **[65]	-	5.6 (5.1-6.0) (*n*=4)	1/2 Yes 1/2 Yes with modifications	1/2 High 1/2 Satisfactory
**APTA 2012 **[56]	55 [44–65, 86, 87] (*n*=3)	4.8 (*n*=2)	2/3 Yes with modifications 1/3 Raw	3/3 Low
**KCE 2017 **[43]	83.3 [41, 61–81, 86–103] (*n*=3)	5 (4.5-6) (*n*=3)	3/4 Yes 1/4 Yes with modifications	3/4 High 1/4 Moderate
**CCGPP 2016 **[55]	44 (29.2-47) (*n*=3)	5 (*n*=2)	1/3 Yes with modifications 1/3 No 1/3 Raw	2/2 High

OA1, Overall assessment 1; OA2, Overall assessment 2

^a^Frequency of ratings across appraisals (e.g., 3 out of 5 appraisals judged “Yes”)

Raw*, raw recommendations for use* within the same appraisal (e.g., one rater in NG 2021 judged “Yes” and one judged “yes with modification”

*ACP* American College of Physicians, *APS* American Pain Society, *APTA* American Physical Therapy Association, *CCGPP* Council on Chiropractic Guidelines and Practice Parameters, *KCE* Belgian Health Care Knowledge Centre, *NICE* National Institute for Health and Care Excellence

### Recommended CPGs

Additional file 3 lists the CPGs that can be recommended for clinicians based on: overall assessment 2 (i.e., yes recommendation for use); quality rating (i.e., high); agreement of appraisals that overlapped for the same CPG (i.e., perfect agreement as measured by the ICC); and updated status of publication. Overall, NICE 2016 [63] and CCGPP 2016 [55] ranked first and second, respectively.

## Discussion

More than one third of CPGs for LBP have been re-assessed by different appraisals in the last six years. This implies a potential waste of time and resources, since many appraisals assessed the same CPGs. Researchers contemplating AGREE II appraisal of CPGs for LBP should carefully think before embarking on a new systematic review and editors should bear in mind that much has already been published. Although the PRISMA [86] and the PROSPERO [87, 88] initiatives have been around for more than 10 years, half (53%) of the appraisals were registered as systematic reviews. Nonetheless, perfect/substantial agreement in 78% of AGREE II ratings confirmed the CPG quality. Agreement was highest in the ACP 2017 [47], the APTA 2012 [56], and the APS 2009 [65], and lowest in the NICE 2009 [64], the TOP 2017 [53], and the ASIPP 2013 [49].

Here we compared similarities and differences across appraisals. A plausible explanation for the discrepancy in the degree of agreement on CPGs is that the AGREE II tool includes different information within a single item. Raters may focus their attention on some aspects more than others because there is no composite weight of judgement [55]. In addition, discordances may stem from the availability and ease of access to supplementary contents to better address domain judgment. AGREE II does, however, recommend that raters read the clinical CPG document in full, as well as any accompanying documents [9].

Analysis of variability within domains of the appraisals that assessed the same CPG showed that the two most variable domains were Domain 6 – Editorial Independence and Domain 5 – Applicability and Domain 2 – Stakeholder Involvement. There was poor reporting for some CPGs in Domain 6 item scores, resulting in potential financial conflict of interest between CPG developers, stakeholders, and industry [89]. Conflict of interest can arise for anyone involved in CPG development (funders, systematic review authors, panel members, patients or their representatives, peer reviewers, researchers) [90] and have an impact on biased recommendations with consequences for patients [91, 92]. Affiliation, member role, and management of potential conflict of interest in the recommendation process must be transparently reported to improve judgment consistency. There is an important difference between declaring an interest and determining and managing a potential conflict of interest [93, 94]. While not all interests constitute a potential cause for conflict, assessment must be fully described before taking a decision [95]. Furthermore, inadequate information results in an unclear conflict of interest statement, which can open the way to subjective judgment and variation in the scores for this domain. One solution would be to have a document that identifies explicit links between interests and conflict of interst for each CPG recommendation, so as to give a transparent judgment.

Unsurprisingly, Domain 2 – Stakeholder Involvement also varied widely because it shares the same issue of the description of CPG development groups. This domain presents broad assessment of patient values, preferences, and experiences (e.g., patients/public participation in a CPG development group, external review, interview or literature review), which could be perceived as valid alternative strategies and not a combination of actions. For example, one would expect patient involvement on a LBP CPG development panel rather than consultation of the literature on patient values. This choice reflects patient involvement because it influences guideline development, implementation, and dissemination. CPGs developed without patient involvement may ultimately not be acceptable for use [96].

Domain 5 – Applicability was found poorly and heterogeneously reported in other conditions, too [5, 97]. One reason for domain variability is that the items in this domain often rely on information supplementary to the main guideline document. Supplementary documents may sometimes no longer be retrievable, especially if the CPG is outdated. Implementation of CPGs is not always considered an integrated activity of CPG development. Without an assessment of CPG uptake (e.g., monitoring/audit, facilitators, barriers to its application), its recommendations may not be fully and adequately translated into clinical practice [5]. In some cases, monitoring is not enough without indications or solutions to overcome barriers. Balancing judgments is difficult and may result in variability for this domain.

Finally, due to missing data (overall assessment 1 not reported in 35% of appraisals; overall assessment 2 not reported in 76% of appraisals) and heterogeneity of reporting (1-7 point or 0-100 point scales; final recommendation for use or raw recommendation for use), we found it difficult to synthesize agreements and provide implications for clinical practice. Though not mandatory in the AGREE II tool, a quality rating (high, moderate, low) was reported in 53% of appraisals but agreement was perfect in only half of the appraisals. Our findings are consistent with a previous study on CPG appraisals in rehabilitation in which reporting of the two overall assessments was poor and the quality ratings differed from low to high in more than one fourth of approaisals when different cut-offs were applied to rate the same CPG [21].

In general, variability can be partly explained by the different number of items in each domain, the number of raters, and the subjective rating of AGREE II items that can be differently weighted as leniency and strictness bias [98].

Another factor that could explain variability is the suboptimal use of the AGREE II tool: 65% of the CPG appraisals in our sample did not provide information on whether the raters had received training in use of the AGREE II tool [99] and only 18% involved at least four raters, as recommended in the AGREE II manual [9]. Clinical and methodological competences should always be well balanced among raters,and reported to ensure adherence to high standards. We strongly suggest appraisals report whether raters have received AGREE II training [99]. Some issues with AGREE II validity may arise (e.g., AGREE II video tutorials; “My AGREE PLUS” platform) [100] when the training resources are not consistently updated.

### Strengths and limitations

This is the first meta-epidemiological study to examine the overlapping of appraisals applying the AGREE II tool to CPGs for LBP. The sizeable sample of appraisals encompassing CPGs for LBP prevention, diagnosis, and treatment supports the external validity of our findings. Nevertheless, some limitations must be noted. We used as the unit for analysis the overlapping CPGs assessed by at least three appraisals, including CPGs assessed by up to eight appraisals, which may have increased judgment variability. On the conservative side, however, when we restricted our analysis to CPGs assessed by at least five appraisals, the results showed patterns similar to the larger primary sample. We then assessed the variability of overall assessments and quality ratings reported by appraisals when the data were available and homogeneously reported. We did not standardize or convert judgments when the data were reported heterogeneously (e.g., 1-7 point scale or 0-100 scale; final recommendation or raw recommendation for use). This cautious strategy meant that we could not measure the variability of overall assessments for the whole sample since the data were missing from 35% (overall assessment 1) to 76% (overall assessment 2) of appraisals. The percentages of poor reporting are known [97, 101, 102] and similar findings were documented for a large sample of CPGs on rehabilitation (35% overall assessment 1 and 58% overall assessment 2) [21].

### Implications

We suggest that time and resources in conducting LBP appraisals can be optimized when appraisal raters follow the AGREE II manual recommendations for conducting (e.g., number of raters; AGREE II training) and reporting (e.g., overall assessment 2). Before starting a new appraisal, researchers should check academic databases and systematic review registers (e.g., PROSPERO) for published appraisals. Also journal editors could help reduce redundancy by checking compliance with the AGREE II manuals and high-quality standards of reporting for manuscript submissions. Finally, the AGREE Enterprise should invest efforts to promote more transparent and detailed reporting (i.e., support of judgment for AGREE II domains and overall assessments). Considering a wide evaluation including overall assessment 2 (i.e., yes recommendation for use), quality rating (i.e., high), agreement of appraisals that overlapped for the same CPG (i.e., perfect agreement) and updated status of publication, we found that NICE 2016 [63] and CCGPP 2016 [55] would be of value and benefit to clinicians in their practice with LBP patients.

We are aware that a CPG has a limited life span between systematic search strategy to answer the clinical questions and year of publication of the guideline itself [27]. The validity of recommendations more than three years old is often potentially questionable [103].

## Conclusion

We found that more than one third of the CPGs in our sample had been re-assessed for quality by multiple appraisals during the last six years. We found poor and heterogeneous reporting of recommendations for use (i.e., overall assessment 2), which generates unclear information about their application in clinical practice. Clinicians need to be able to rely on high quality CPGs based on updated evidence with perfect agreement by multiple appraisals.

## Supplementary Information

**Additional file 1. **Search strategy.

**Additional file 2.** Exclusion criteria of appraisals.

**Additional file 3.** List of CPGs recommend considering quality ratings, OA2, ICC and status of publication.

## Data Availability

The datasets generated and analysed during the current study are available in the OSF repository, https://osf.io/rz7nh/.

## Abbreviations

ACOEM: American College of Occupational and Environmental Medicine
ACP: American College of Physicians
AGREE II: Appraisal of Guidelines for Research & Evaluation II
ACR: American College of Radiology
AOA: American Osteopathic Association
APS: American Pain Society
APTA: American Physical Therapy Association
ASIPP: American Society of Interventional Pain Physicians;
BPS: British Pain Society
CAAM: China Association of Acupuncture-Moxibustion
CCGI: Canadian Chiropractic Guideline Initiative
CI: Confidence Interval
CPGs: Clinical Practice Guidelines
CCGPP: Council on Chiropractic Guidelines and Practice Parameters
DAI: Deutsches Ärzteblatt International
DHA: Danish Health Authority
DSA: Dutch Society of Anesthesiologists
IQR: Interquartile Range
ICC: Intraclass correlation
ICSI: Institute for Clinical Systems Improvement
IRR: Inter-rater reliability
KCE: Belgian Health Care Knowledge Centre
KIOM: Korea Institute of Oriental Medicine
KNGF: Koninklijk Nederlands Genootschap voor Fysiotherapie
LBP: Low Back Pain
MSK: Musculoskeletal
NASS: North American Spine Society
NICE: National Institute for Health and Care Excellence
NVL: Nationale Versorgungs Leitlinie
OA1: Overall assessment 1
OA2: Overall assessment 2
OMG: Ottawa Methods Group
PARM: Philippine Academy of Rehabilitation Medicine
PEDro: Physiotherapy Evidence Database
PSP: Polish Society of Physiotherapy
PRISMA: Preferred Reporting Intervention for Systematic Review and Meta-analysis
Q: Quartile
SIGN: Scottish Intercollegiate Guidelines Network
SOEGCP: State of Oregon Evidence-based Clinical Guidelines Project
TOP: Toward Optimized Practice Low Back Pain Working Group
VADoD: Veterans Affairs/Department of Defense Collaboration Office
