# Supplementary material for: More than one third of clinical practice guidelines on low back pain overlap in AGREE II appraisals. Research wasted?
Source: BMC Med Res Methodol. 2022 Jul 5;22:184. doi: 10.1186/s12874-022-01621-w (PMC9254584; doi:10.1186/s12874-022-01621-w)
Supplement: Supplementary file 1 — Additional file 1. Search strategy. [file 12874_2022_1621_MOESM1_ESM.docx]

## **Appendix 1.**

Search strategy PUBMED

(back pain[MeSH Terms] OR “low back pain” [All fields] OR “back pain”[All fields] OR backache [All fields] OR “lumba*”[All fields] OR “back disorder”[All fields]) AND ("agree"[tiab] OR “Appraisal of Guidelines for Research & Evaluation Instrument” OR "apprais*"[tiab]) AND ("guideline"[Publication Type] OR "guidelines as topic"[MeSH Terms] OR "guideline"[tiab] OR "practice guideline"[Publication Type] OR "practice guidelines as topic"[MeSH Terms] OR "clinical practice guideline"[tiab])

Limit 2010-2021
