# Supplementary material for: More than one third of clinical practice guidelines on low back pain overlap in AGREE II appraisals. Research wasted?
Source: BMC Med Res Methodol. 2022 Jul 5;22:184. doi: 10.1186/s12874-022-01621-w (PMC9254584; doi:10.1186/s12874-022-01621-w)
Supplement: Supplementary file 2 — Additional file 2. Exclusion criteria of appraisals. [file 12874_2022_1621_MOESM2_ESM.docx]

**Additional File 2. Exclusion criteria of appraisals**

| APPRAISAL | EXCLUSION CRITERIA |
| --- | --- |
| Acevedo 2016(25) | Single intervention, management or pathology related to lumbar pain, management of neoplasms, infection, trauma, and spinal fracture. Reviews without evidence-based recommendations |
| Anderson 2021 (26) | Other spinal conditions |
| Castellini 2020(27) | CPG not primarily focused on LBP; CPG not issued by a national or international society; consensus statements, systematic reviews or commentary editorials related to published CPGs; interventions other than therapeutic (e.g., prevention, diagnosis); population subgroups (e.g., pregnant women), specific causes (e.g., spondylarthritis) or mixed/generic population (e.g., musculoskeletal chronic pain). |
| Corp 2021 (28) | Non-European CPGs; not evidence based CPG; pediatric population; result of severe trauma (fracture and spinal cord injury): inflammatory arthritis including spondyloarthropathy; (chronic) pain that may encompass spinal pain; secondary care with an established diagnosis of radiculopathy; surgical treatment options/comparisons or specific interventions not limited to spinal pain; populations admitted to hospital (not ambulatory care) |
| Doniselli 2018(29) | CPG not primarily focused on LBP, such as national/ international CPGs in which LBP was briefly mentioned in the context of a more comprehensive disease evaluation. |
| Ernstzen 2017(30) | Only chronic pain of non-MSK origin (e.g., sickle cell disease, neuropathic pain, malignancy); CPGs focused solely on secondary, tertiary or specialist healthcare settings |
| Franz 2015(31) | Multidisciplinary treatment, prevention, and promoting health, only manual therapy |
| Hoydonckx 2020(32) | CPGs derived entirely from another CPG, multiple reports of the same CPG; reviews with recommendations made by a single author and recommendations for non-procedural approaches; health technology assessments. |
| Krenn 2020(33) | Infection, tumor, osteoporosis, fracture, metabolic disease, inflammatory arthritis |
| Lin 2020(34) | Traumatic MSK pain, single treatment modality (e.g., surgery), traditional healing/ medicine, specific disease processes (e.g., inflammatory arthropathies) or those that were private for-profit and required payment to access. |
| Meroni 2019(35) | CPG for management of acute LBP, occupation-related LBP, secondary care or prevention of LBP developed by one individual or one regional health care center/hospital; copied or summarized another including CPG; comprised a single report or article on CPG evaluation/; narrative review; provided recommendations only for specific approaches (e.g., massage, manipulation, physical modalities, etc.) without addressing primary care management. |
| Ng 2021(36) | Not developed by non-profit organizations (e.g., academic institutions, government agencies, disease-specific foundation, professional associations or societies); non-English language; consensus statements |
| Nordin 2018(37) | CPGs without evidence-based recommendations for assessment or diagnosis (e.g., CPGs for treatment only); CPGs developed solely on the basis of consensus; CPGs that do not report the methods used to develop recommendations; facet joint injections and epidural injections were excluded. |
| Rathbone 2020(38) | LBP primarily related to cancer, fracture, infection, inflammatory diseases or other serious conditions |
| Stander 2020*(39) | CPGs not written in the last 5 years; not-English language |
| Wong 2017(40) | CPG that did not include treatment recommendations; summary or copy of previous CPGs; consensus opinion; no systematic literature search or critical appraisal of studies to derive recommendations; only targeted invasive interventions (e.g., injection, surgery) |
| Yaman 2015(41) | - |

**Legend:** LBP, low back pain, CPG, clinical practice guideline, MSK, musculoskeletal
