# Supplementary material for: More than one third of clinical practice guidelines on low back pain overlap in AGREE II appraisals. Research wasted?
Source: BMC Med Res Methodol. 2022 Jul 5;22:184. doi: 10.1186/s12874-022-01621-w (PMC9254584; doi:10.1186/s12874-022-01621-w)
Supplement: Supplementary file 3 — Additional file 3. List of CPGs recommend considering quality ratings, OA2, ICC and status of publication. [file 12874_2022_1621_MOESM3_ESM.docx]

**Additional File 3. List of CPGs recommended considering quality ratings, OA2, ICC and status of publication.**

| CPG | year | Mean quality ratings | Mean OA2 | ICC | Ranking |
| --- | --- | --- | --- | --- | --- |
| NICE 2016 (63) | 2016 | High | Yes | Perfect | 1 |
| CCGPP 2016 (Globe) (55) | 2016 | High | Yes mods | Perfect | 2 |
| ICSI 2018 (Thorson) (44) | 2018 | Moderate | Yes mods | Perfect | 3 |
| ACP 2017 (Qaseem) (47) | 2017 | Moderate | Yes mods | Perfect | 3 |
| DHA 2017 (Stochkendahl) (45) | 2017 | Moderate | Yes mods | Perfect | 3 |
| VaDod 2017 (Pangarkar) (48) | 2017 | Moderate | Yes mods | Perfect | 3 |
| KCE 2017 (Van Wambeke) (43) | 2017 | High | Yes | Substantial | 4 |
| APS 2009 (Chou) (65) | 2009 | High | Yes mods | Perfect | 5 |
| NASS 2014 (Kreiner) (51) | 2014 | Moderate | Yes mods | Perfect | 6 |
| CCGI 2018 (Bussieres) (60) | 2018 | High | Yes | Moderate | 6 |
| SIGN 2013 (62) | 2013 | High | Yes* | Substantial | 7 |
| CAAM 2016 (Zhao) (42) | 2016 | Low | Yes mods | Perfect | 8 |
| DAI 2017 (Chenot) (58) | 2017 | Moderate | Yes mods | Moderate | 9 |
| OMG 2012 (Brusseau) (61) | 2012 | Low | Yes mods | Perfect | 10 |
| APTA 2012 (Delitto) (56) | 2012 | Low | Yes mods | Perfect | 10 |
| Cheng 2012 (59) | 2012 | Low | Yes mods | Perfect | 10 |
| NASS 2013 (Kreiner) (50) | 2013 | Moderate | Yes mods | Substantial | 11 |
| KNGF 2013 (Staal) (46) | 2013 | Moderate | No | Perfect | 12 |
| NICE 2009 (64) | 2009 | High | Yes mods | Fair | 12 |
| ASIPP 2013 (Manchikanti) (49) | 2013 | Moderate | Yes mods | Slight | 13 |
| ICSI 2012 (Goertz) (54) | 2012 | Low | Yes mods | Substantial | 14 |
| TOP 2017 (53) | 2017 | Moderate | No | Slight | 15 |
| PSP 2017 (Kassolik) (52) | 2017 | Low | No | Substantial | 16 |

**Legend:** ACP: American College of Physicians; APS: American Pain Society; APTA: American Physical Therapy Association; ASIPP: American Society of Interventional Pain Physicians; CAAM: China Association of Acupuncture-Moxibustion; CCGI: Canadian Chiropractic Guideline Initiative; CCGPP: Council on Chiropractic Guidelines and Practice Parameters; CPG: Clinical Practice Guideline; DAI: Deutsches Ärzteblatt International; DHA: Danish Health Authority; ICC: Intraclass Correlation Coefficient; ICSI: Institute for Clinical Systems Improvement; KCE: Belgian Health Care Knowledge Centre; KNGF: Koninklijk Nederlands Genootschap voor Fysiotherapie; NASS: North American Spine Society; NICE: National Institute for Health and Care Excellence; OA2: Overall Assessment 2; OMG: Ottawa Methods Group; PSP: Polish Society of Physiotherapy; SIGN: Scottish Intercollegiate Guidelines Network; TOP: Toward Optimized Practice Low Back Pain Working Group; VADoD: Veterans Affairs/Department of Defense Collaboration Office.

*imputed data considering quality ratings
